# Rotavirus vaccine impact and socioeconomic deprivation: an interrupted time-series analysis of gastrointestinal disease outcomes across primary and secondary care in the UK

**DOI:** 10.1186/s12916-017-0989-z

**Published:** 2018-01-29

**Authors:** Daniel Hungerford, Roberto Vivancos, Jonathan M. Read, Miren Iturriza-Gόmara, Neil French, Nigel A. Cunliffe

**Affiliations:** 1 The Centre for Global Vaccine Research, Institute of Infection and Global Health, University of Liverpool, L69 7BE Liverpool, UK; 2 Field Epidemiology Services, Public Health England, L3 1DS Liverpool, UK; 3 NIHR Health Protection Research Unit in Gastrointestinal Infections, L69 3GL Liverpool, UK; 4 NIHR Health Protection Research Unit in Emerging and Zoonotic Infections, L69 3GL Liverpool, UK; 5 Centre for Health Informatics, Computing and Statistics, Lancaster Medical School, Faculty of Health and Medicine, Lancaster University, Lancaster, LA1 4YG UK; 6 Department of Microbiology, Alder Hey Children’s NHS Foundation Trust, L12 2AP Liverpool, UK

**Keywords:** Surveillance, Rotavirus, Vaccine, Gastroenteritis, Paediatric, Diarrhoea, Health equity, Socioeconomic inequalities, Health service, Epidemiology

## Abstract

**Background:**

Rotavirus causes severe gastroenteritis in infants and young children worldwide. The UK introduced the monovalent rotavirus vaccine (Rotarix®) in July 2013. Vaccination is free of charge to parents, with two doses delivered at 8 and 12 weeks of age. We evaluated vaccine impact across a health system in relation to socioeconomic deprivation.

**Methods:**

We used interrupted time-series analyses to assess changes in monthly health-care attendances in Merseyside, UK, for all ages, from July 2013 to June 2016, compared to predicted counterfactual attendances without vaccination spanning 3–11 years pre-vaccine. Outcome measures included laboratory-confirmed rotavirus gastroenteritis (RVGE) hospitalisations, acute gastroenteritis (AGE) hospitalisations, emergency department (ED) attendances for gastrointestinal conditions and consultations for infectious gastroenteritis at community walk-in centres (WIC) and general practices (GP). All analyses were stratified by age. Hospitalisations were additionally stratified by vaccine uptake and small-area-level socioeconomic deprivation.

**Results:**

The uptake of the first and second doses of rotavirus vaccine was 91.4% (29,108/31,836) and 86.7% (27,594/31,836), respectively. Among children aged < 5 years, the incidence of gastrointestinal disease decreased across all outcomes post-vaccine introduction: 80% (95% confidence interval [CI] 70–87%; *p* < 0.001) for RVGE hospitalisation, 44% (95% CI 35–53%; *p* < 0.001) for AGE hospitalisations, 23% (95% CI 11–33%; *p* < 0.001) for ED, 32% (95% CI 7–50%; *p* = 0.02) for WIC and 13% (95% CI -3–26%; *p* = 0.10) for GP. The impact was greatest during the rotavirus season and for vaccine-eligible age groups. In adults aged 65+ years, AGE hospitalisations fell by 25% (95% CI 19–30%; *p* < 0.001).

The pre-vaccine risk of AGE hospitalisation was highest in the most socioeconomically deprived communities (adjusted incident rate ratio 1.57; 95% CI 1.51–1.64; *p* < 0.001), as was the risk for non-vaccination (adjusted risk ratio 1.54; 95% CI 1.34–1.75; *p* < 0.001). The rate of AGE hospitalisations averted per 1,000 first doses of vaccine was higher among infants in the most deprived communities compared to the least deprived in 2014/15 (28; 95% CI 25–31 vs. 15; 95% CI 12–17) and in 2015/16 (26; 95% CI 23–30 vs. 13; 95% CI 11–16).

**Conclusions:**

Following the introduction of rotavirus vaccination, incidence of gastrointestinal disease reduced across the health-care system. Vaccine impact was greatest among the most deprived populations, despite lower vaccine uptake. Prioritising vaccine uptake in socioeconomically deprived communities should give the greatest health benefit in terms of population disease burden.

**Electronic supplementary material:**

The online version of this article (doi:10.1186/s12916-017-0989-z) contains supplementary material, which is available to authorized users.

## Background

Prior to the introduction of rotavirus vaccination, rotavirus was the leading cause of severe gastroenteritis in children under 5 years of age worldwide, resulting in approximately 453,000 deaths per year and 40% of diarrhoeal hospital admissions [1, 2]. Two orally administered live-attenuated rotavirus vaccines, Rotarix® (GlaxoSmithKline Biologicals, Belgium) and RotaTeq® (Merck Vaccines, USA), have been introduced in over 90 countries worldwide [3]. The global mortality from rotavirus gastroenteritis (RVGE) has subsequently more than halved (recently estimated at between ~120,000 and ~215,000) and the number of all-cause acute gastroenteritis (AGE) hospitalisations is estimated to have reduced by 38% [4–7].

Although the majority of the severe disease burden is in developing countries, rotavirus was estimated to cause approximately 80,000 general practice (GP) consultations and 750,000 diarrhoea episodes each year in the UK [8]; 45% of hospitalisations and 20% of emergency department (ED) attendances for AGE in children under 5 years of age were attributable to rotavirus [9]. The National Health Service (NHS) in England is free at the point of use for all UK residents, with vaccinations included in the routine immunisation schedule also free of charge. The monovalent rotavirus vaccine (Rotarix®) was introduced into the UK childhood immunisation schedule in July 2013, with two doses delivered at 8 and 12 weeks of age [10]. Vaccine uptake in England increased rapidly, reaching over 91% for one dose by February 2014 and over 94% by mid-2016 [11]. To date, studies in the UK have separately, and for varied populations and time periods, analysed vaccine impact on rotavirus laboratory detections (77% reduction in infants) [12], RVGE hospitalisations (> 80% reduction in infants) [13], all-cause AGE hospitalisations (26% in infants) [12] and GP attendances for diarrhoea related illness (20–30% in those under 5 years old) [14].

This study aimed to assess the effect of rotavirus vaccination on multiple levels of the UK health-care system simultaneously, by examining the trends in hospitalisations, ED attendances, community health consultations and GP consultations for outcomes of gastroenteritis, diarrhoea and rotavirus gastroenteritis in a defined population before and after vaccine introduction. This approach will, for the first time, provide estimates of rotavirus vaccine impact in an entire health economy. Secondly, within the UK, children under 5 years of age are over-represented in the most socioeconomically deprived populations [15, 16], and experience significantly higher incidence of all-cause AGE hospitalisations than more affluent populations [17]. It is known that in the UK, the uptake of routine childhood vaccines (e.g. vaccines for measles, mumps and rubella, human papillomavirus and influenza) is lower in socioeconomically deprived populations [18–20]. Thus, we examined the uptake and impact of rotavirus vaccination in Merseyside, an area with a wide variation in socioeconomic deprivation, to assess whether vaccine uptake and impact are equitable.

## Methods

### Study setting

The study population was the metropolitan area of Merseyside, England, with an estimated resident population of 1.4 million and an annual birth cohort of approximately 16,000. In 2016, 80,000 of the population were under 5 years of age [16]. Merseyside contains five local authorities (Knowsley, Liverpool, Sefton, St Helens and Wirral), containing multiple NHS trusts and organisations. Health-care for the population is provided in the community by GP practices and walk-in centres (WICs), offering both primary and urgent care. There are five hospitals with emergency and secondary-care facilities, including a large paediatric hospital (Alder Hey Children’s NHS Foundation Trust). The organisations and facilities have been previously described [21].

### Data sources and case definitions for outcome measures

Data sources and full case definitions have been previously published [21]. Table 1 summarises these details, and amends any discrepancies. Notably, data on GP consultations were obtained through the NHS clinical commissioning groups (CCGs). Coding for non-infectious gastroenteritis (ICD-10 K52.9) was included in the all-cause AGE hospitalisation outcome measure, since unspecified gastroenteritis was classified under this code until April 2012 [22].

**Table 1:** Details of each outcome measure and data source

Data source	Population	Outcome	Denominator/offset	Age in months (m) or years (y)	Time period
Alder Hey Children's NHS Foundation Trust	RVGE hospitalisations. Alder Hey's footprint covers the majority of Merseyside children	Laboratory-confirmed rotavirus gastroenteritis. Rotavirus antigen detected by immunochromatography test (2005–2009) or by enzyme immunoassay (2002–2005 and 2009 onwards) in a faecal specimen of a child with acute gastroenteritis	Total hospitalisations per month by age group	0–14 y: < 12 m; 12–23 m; 24–59 m; 5–14 y	July 2002 to June 2016
Hospital Episode Statistics – admitted patient care	Merseyside residents attending any hospital in England	Hospitalisation for all-cause acute gastroenteritis. Identified by ICD-10 codes: A00–A09) or as non-infectious gastroenteritis (K52.9), in any diagnosis field	Yearly estimated age-specific population of Merseyside. Source: Office for National Statistics; accessed through Public Health England [16]	All ages: < 12 m; 12–23 m; 24–59 m; 5–14 y; 15–64 y; 65+	July 2000 to June 2016
Hospital Episode Statistics – accident and emergency	Merseyside residents attending three major emergency departments in Merseyside	Emergency department attendance for gastrointestinal conditions (AE diagnosis code 26); excluding subsequent admissions. Missing diagnosis data was imputed for one emergency department between November 2010 and March 2011	Total emergency department attendances (excluding subsequent admissions) per month by age group	All ages: < 12 m; 12–23 m; 24–59 m; 5–14 y; 15–64 y; 65+	July 2008 to June 2016
Walk-in centre attendance records	Attendances at walk-in centres in Wirral, covering an estimated resident population of 320,000	Walk-in centre attendance for infectious gastroenteritis. Read Codes: gastroenteritis – presumed infectious origin (A0812), diarrhoea of presumed infectious origin (A083); infantile viral gastroenteritis (A07y1); infectious gastroenteritis (A0803); enteritis due to rotavirus (A0762); and infectious diarrhoea (A082)	All walk-in centre attendances per month by age group	All ages: < 12 m; 12–23 m; 24–59 m; 5–14 y; 15–64 y; 65+	July 2011 to June 2016
GP records	Consultations at 136 GP practices in Merseyside, covering an estimated population of 790,000	Consultations for infectious gastroenteritis (Read Codes as above for walk-in centre)	Yearly estimated GP registered population by age group. Data were available from 2010 to 2016, therefore estimates for 2007/2008 and 2008/2009 were synthetically estimated using predictions from linear regression models. Source: Public Health England and participating GP practices	All ages: < 12 m; 12–23 m; 24–59 m; 5–14 y; 15–64 y; 65+	July 2007 to June 2016

*GP* general practice, *RVGE* rotavirus gastroenteritis, *AE* accident and emergency

### Area of residence and socioeconomic deprivation

In each of the health data sets accessed, an indicator for neighbourhood area of residence (lower super output area [LSOA]) was included. English LSOAs are small statistical boundaries defined following the 2001 and 2011 censuses and consist of approximately 1,500 people. A standardised measure of socioeconomic deprivation was assigned to each participant, using the LSOA of their residence and the English indices of deprivation 2015, the Index of Multiple Deprivation (IMD) [15]. The English indices of deprivation are produced and quality controlled using national census and other administrative data [15]. They are constructed from 37 robust indicators in seven domains: education skills and training, employment, income, living environment, crime, and barriers to housing and other services [15]. These domains are combined and weighted to calculate one of the most robust and commonly used measures of deprivation in England, the IMD [15, 18].

### Uptake of rotavirus vaccination

Pseudo-anonymised vaccine status data were extracted from the Child Health Information Service (CHIS) [23, 24], which is managed locally by NHS trusts and holds a unique record for each child born in these areas until the age of 18 years. We obtained a CHIS data extract on children eligible for rotavirus vaccination born from May 2013 to June 2016. The extract included a unique identifier, year and month of birth, year and month of first and second doses of rotavirus vaccine, and LSOA of residence. CHIS could be accessed for four out of the five local authorities in Merseyside. Data for Wirral could not be extracted due to the lack of access to the CHIS database during the study period, which was related to organisational restructuring. We used codes in the CHIS data set to exclude from the analysis deaths, stillbirths and children who were born in Merseyside during the study period but subsequently moved out.

### Statistical analyses

#### Impact

We examined monthly hospitalisations and attendances to health-care providers using an interrupted time-series design. Firstly, to predict counterfactual numbers of hospitalisations and attendances that would have been expected in the absence of vaccination for the vaccine period, we fitted generalised linear models with Poisson or negative binomial distributions (to account for over-dispersion in the data) to pre-vaccine introduction monthly counts, offset for a data-set-specific denominator (Table 1). We adjusted for seasonal trends by including a categorical term for calendar month and secular trends by including a linear term for surveillance year (July to June) as explanatory variables in the models. Secondly, to quantify the percentage reduction in monthly attendances and hospitalisations, we included all data pre- and post-vaccine introduction in a second model with a binary indicator variable denoting the post-vaccine period. This second model also included the same terms to adjust for seasonal and secular trends and allowed the calculation of incidence rate ratios (IRRs). The percentage reduction was calculated as $100\times \left(1-\mathrm{IRR}\right)$. The RVGE season in the UK in the pre-vaccine period was consistently between the months of January and May with the peak occurring in early to mid-March in most years [25]. For the sensitivity analysis, we examined the specificity of the end point by stratifying by events that occurred in-season (January to May) and out-of-season (June to December). To investigate vaccine impact by age, the analysis was stratified by age group (< 12 months, 12–23 months, 24–59 months, 5–14 years, 15–64 years, 65+ years and 0–59 months).

#### Socioeconomic deprivation, vaccine uptake and hospitalisations

Firstly, we wished to assess whether the incidence of all-cause AGE hospitalisations varied by level of socioeconomic deprivation. To achieve this, we fitted negative binomial generalised linear models with the number of hospitalisations as the dependent variable and the quintile of deprivation as the independent variable, offset for population denominator and adjusting again for seasonal and secular trends. The quintile of deprivation was calculated using the IMD scores for LSOAs nationally, whereby quintile 5 is the least deprived and quintile 1 the most deprived. Since the population of Merseyside is skewed towards the most deprived national quintiles (45% of the population are in the most deprived quintile and 8% in the least deprived), we combined the two least deprived quintiles into category 4/5 (least deprived). All-cause AGE hospitalisations were included in the model for the time period July 2004 to June 2016 because LSOA information was not available prior to April 2004. The models allowed the calculation of IRR for socioeconomic deprivation groups by comparing the 4/5 least deprived category to the other quintiles, stratified by age group.

Secondly, we describe the uptake of the first and second doses of rotavirus vaccine by month of birth for children born between May 2013 and December 2015. December 2015 was selected as the cut-off to allow all children in the cohort to reach 25 weeks of age, the upper time limit for rotavirus vaccination [26]. To investigate associations between socioeconomic deprivation and vaccine uptake, we fitted logistic regression models where the dependent variable was vaccine status and the independent variable was the national quintile of IMD and adjusted for gender and year and month of birth. The models allowed the calculation of risk ratios (RRs) for socioeconomic deprivation group by comparing the 4/5 least deprived category to the other quintiles.

Finally, we estimated the all-cause AGE hospitalisations averted per 1,000 vaccine first doses delivered in the 2014/15 and 2015/16 seasons for vaccine-eligible cohorts aged < 12 months and 12–23 months. We define the rate of hospitalisations averted per 1,000 vaccine first doses delivered as:

$$ {\mathrm{RDA}}_{ijk}=\frac{X_{ijk}-{Y}_{ijk}}{P_{ijk}{V}_{ijk}} $$

where RDA is the rate of hospitalisations averted per 1,000 vaccine first doses delivered. *X* is the model-predicted counterfactual number of hospitalisations that would have been expected in the absence of vaccination for the vaccine period. *Y* is the observed number of hospitalisations in the vaccine period, *P* the population denominator, *V* the proportion of the population vaccinated with one dose of rotavirus vaccine, *i* the deprivation group, *j* the age group and *k* the surveillance year.

We used the RDA in the Merseyside population in this study to provide an estimate of the number of all-cause AGE hospitalisations averted at a national level if the 95% vaccine uptake targets set by the World Health Organization (WHO) were achieved across all deprivation strata [27, 28]. We define the total number of all-cause AGE hospitalisations averted at a national level in 2015/16 at uniform 95% uptake as:

$$ \mathrm{NDA}=\sum \frac{{\mathrm{RDA}}_{ijk}}{1000}\times \left({N}_{ijk}\times 0.95\right) $$

where NDA is the number of all-cause AGE hospitalisations averted. RDA is the rate of hospitalisations averted per 1,000 vaccine first doses delivered in the Merseyside population. *N* is the national population denominator, derived from mid-year LSOA population estimates 2015/16 [16]. *i* is the deprivation group, *j* is the age group and *k* is the surveillance year.

Data handling and analysis were conducted in R version 3.3 (R Development Core Team, Vienna, Austria).

## Results

### Vaccine uptake

**Fig. 1:**
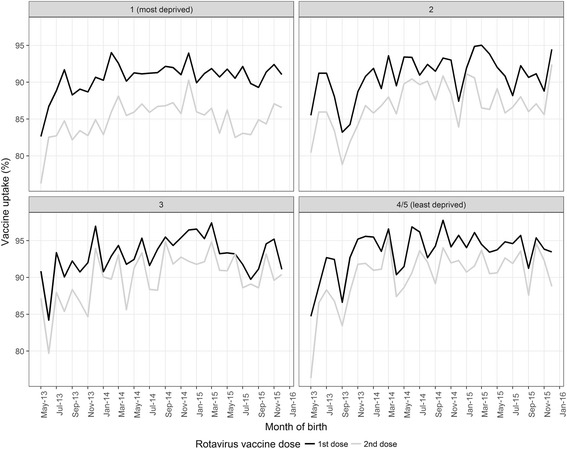
Rotavirus vaccine uptake in 4/5 areas of Merseyside, UK, for children born between May 2013 and December 2015 by deprivation quintile

Rotavirus vaccine uptake (at least one dose of vaccine) in children born between May 2013 and December 2015 was 91.4% (29,108/31,836) and completion of the full rotavirus vaccine schedule (i.e. two doses) was 86.7% (27,594/31,836). In the least deprived population, vaccine uptake for at least one dose was 93.6% (4,135/4,420) and 90.2% (3,989/4,420) for completion of the two-dose schedule; in the most deprived population uptake was 90.6% (16,550/18,259) and 84.9% (15,505/18,259), respectively (Fig. 1). The most deprived populations had a 54% increased risk of non-vaccination compared to the least deprived populations (RR 1.54; 95% CI 1.34–1.75). Furthermore, the most deprived populations had almost twice the risk (RR 1.97; 95% CI 1.62–2.41) of non-completion of the two-dose schedule compared to the least deprived.

### Vaccine impact by age

#### Impact in those under 5 years old

**Fig. 2:**
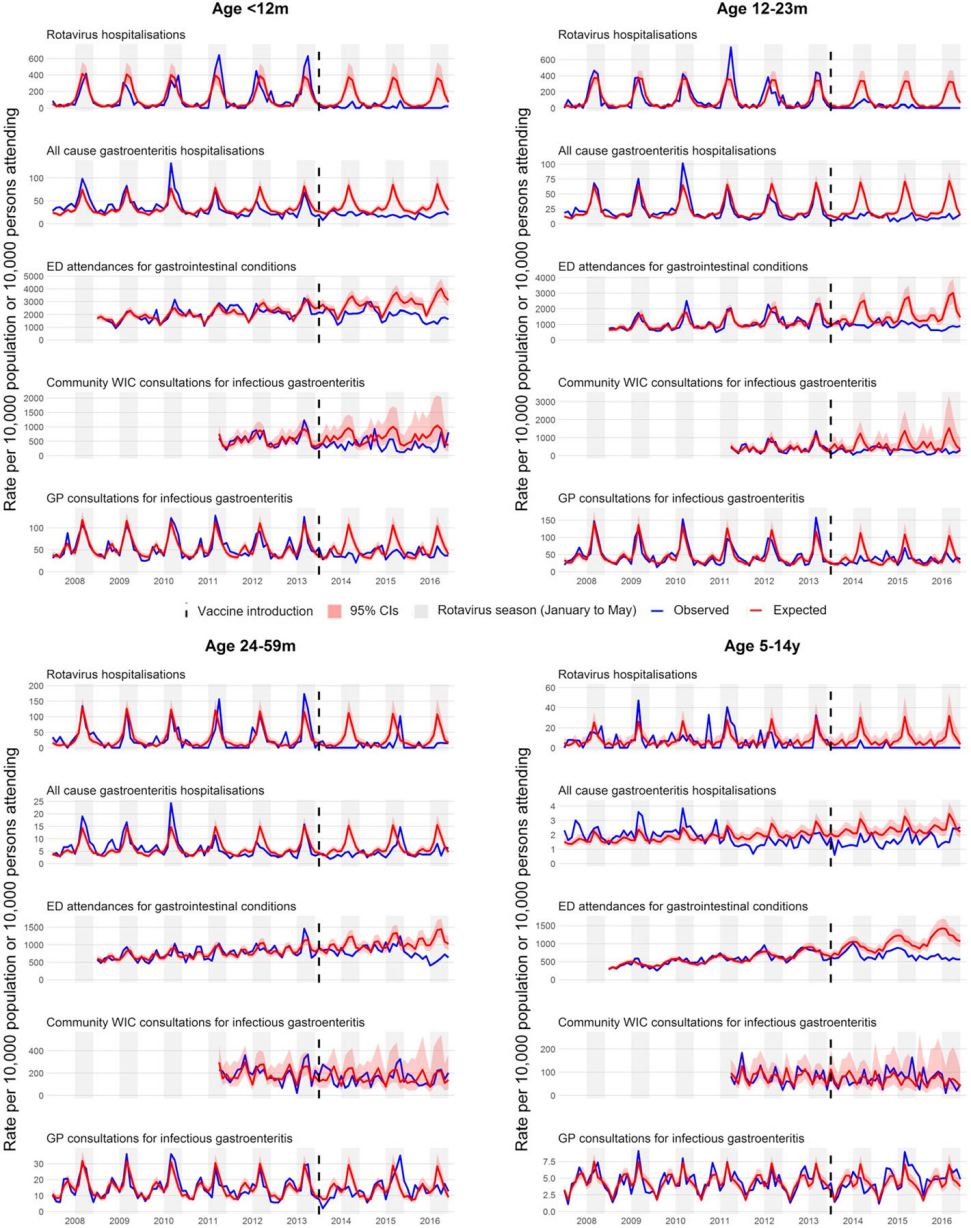
Trends in five study outcome measures for children aged 0–14 years in Merseyside, UK, July 2008 to June 2016. Each analysis examines trends, including a comparison of observed incidence (blue line) after rotavirus vaccination (July 2013 to June 2016) in the UK with expected incidence (red line) and associated 95% confidence intervals (red shaded area) in the absence of vaccination. Expected incidence and 95% confidence intervals are based on predictions from regression models fitted to available historic data for each outcome measure. The black hashed line represents the introduction of rotavirus vaccine in the UK in July 2013. CI confidence interval, ED emergency department, GP general practice, WIC walk-in centre

In children less than 5 years of age, a clearly defined rotavirus season was observed prior to vaccine introduction, with the peak predominately occurring in March across all outcome measures for all years prior to vaccine introduction (Fig. 2). The incidence of gastrointestinal disease fell across all health outcomes following vaccine introduction (Fig. 2 and Table 2). The greatest proportional reduction, 80% (95% CI 70–87%), was for RVGE hospitalisation. All-cause AGE hospitalisations fell by 44% (95% CI 35–53%), ED attendances for gastrointestinal conditions by 23% (95% CI 11–33%), and WIC and GP consultations for infectious gastroenteritis by 32% (95% CI 7–50%) and 13% (95% CI -3–26%), respectively. Reductions were greatest in the rotavirus season for all outcomes. All-cause AGE hospitalisations fell by 58% (95% CI 45–67%) and GP consultations by 29% (95% CI 8–45%).

**Table 2:** Changes in rates of hospitalisation and attendances at different levels of the health system post-rotavirus vaccine introduction in Merseyside, UK

Age group	Mean yearly rate of hospitalisations and attendances (per 10,000)^a^			Percentage reduction in hospitalisation and attendance rates (95% CI)^c^		
	Pre-vaccination	Post-vaccination				
	Observed	Observed	Expected^b^	Full year	January–May	June–December
Hospitalisations for laboratory-confirmed rotavirus to Alder Hey						
< 12 m	129	14	122	87 (78 to 93)	94 (86 to 97)	57 (10 to 81)
12–23 m	123	16	106	84 (73 to 91)	87 (76 to 94)	70 (19 to 91)
24–59 m	33	10	29	66 (44 to 81)	74 (52 to 87)	35 (to 70 to 77)
5–14 y	7	0.3	9	95 (84 to 99)	96 (80 to 99.7)	94 (71 to 99.7)
Total 0–59 m	87	12	81	80 (70 to 87)	88 (80 to 94)	58 (25 to 77)
Hospitalisations for all-cause acute gastroenteritis						
< 12 m	402	230	468	46 (36 to 54)	60 (49 to 69)	35 (20 to 46)
12–23 m	271	128	311	50 (40 to 59)	66 (56 to 74)	37 (19 to 50)
24–59 m	72	54	78	26 (11 to 39)	33 (10 to 50)	22 (1 to 38)
5–14 y	18	20	28	32 (21 to 41)	35 (19 to 48)	29 (13 to 42)
15–64 y	39	60	66	8 (2 to 14)	11 (1 to 19)	6 (1 to 13)
65+	135	157	210	25 (19 to 30)	28 (19 to 36)	22 (15 to 29)
Total 0–59 m	178	104	213	44 (35 to 53)	58 (46 to 67)	35 (22 to 46)
ED attendances for gastrointestinal conditions (no admission)						
< 12 m	2034	1855	2816	22 (11 to 33)	30 (15 to 42)	16 (2 to 29)
12–23 m	1146	892	1650	31 (15 to 43)	41 (19 to 57)	23 (4 to 38)
24–59 m	759	759	1054	12 (-4 to 25)	14 (-15 to 36)	10 (-6 to 24)
5–14 y	552	661	1038	22 (11 to 31)	17 (-2 to 33)	25 (12 to 36)
15–64 y	405	503	993	29 (16 to 40)	30 (4 to 49)	28 (14 to 40)
65+	341	438	788	21 (4 to 34)	25 (-5 to 46)	18 (-3 to 34)
Total 0–59 m	1235	1124	1795	23 (11 to 33)	31 (12 to 45)	18 (4 to 29)
Walk-in centre attendances for infectious gastroenteritis						
< 12 m	574	373	644	37 (6 to 58)	51 (12 to 73)	25 (-26 to 55)
12–23 m	463	256	606	39 (0 to 63)	67 (38 to 83)	5 (-86 to 52)
24–59 m	196	153	167	18 (-20 to 44)	36 (-12 to 64)	-5 (-79 to 38)
5–14 y	79	71	68	0 (-52 to 34)	6 (-77 to 49)	-6 (-86 to 39)
15–64 y	55	51	61	24 (7 to 38)	29 (0 to 49)	21 (-4 to 40)
65+	22	18	52	47 (-15 to 75)	56 (-43 to 86)	38 (-72 to 78)
Total 0–59 m	362	231	363	32 (7 to 50)	51 (22 to 69)	12 (-27 to 39)
GP consultations for infectious gastroenteritis						
< 12 m	674	492	628	19 (4 to 33)	40 (27 to 51)	3 (-20 to 21)
12–23 m	590	418	498	13 (-10 to 31)	38 (11 to 56)	-11 (-44 to 14)
24–59 m	184	166	172	8 (-14 to 26)	7 (-29 to 33)	9 (-20 to 31)
5–14 y	53	56	51	-3 (-21 to 12)	-7 (-38 to 17)	0 (-23 to 19)
15–64 y	41	30	41	26 (18 to 33)	29 (17 to 40)	23 (13 to 32)
65+	35	29	48	36 (25 to 45)	43 (30 to 54)	30 (13 to 43)
Total 0–59 m	363	282	331	13 (-3 to 26)	29 (8 to 45)	0 (-20 to 17)

*CI* confidence interval, *ED* emergency department, *GP* general practice

^a^Table 1 provides specific denominators for each outcome measure

^b^Expected in the absence of vaccination using a negative binomial or Poisson model adjusting for month and rotavirus year for the pre-vaccine years

^c^Percentage change is calculated as 1-IRR. Incidence rate ratio (IRR) was calculated using a negative binomial model or Poisson model adjusting for month and rotavirus year

Disease reductions were highest in vaccine-eligible age groups. RVGE hospitalisation fell by 87% (95% CI 78–93%) in infants aged < 12 months and 84% (95% CI 73–91%) in children 12–23 months. All-cause AGE hospitalisations fell by 46% (95% CI 36–54%) in infants < 12 months and 50% (95% CI 40–59%) in children 12–23 months. For GPs, infectious gastroenteritis consultations fell by 19% (95% CI 4–33%) in infants, averting 136 consultations per 10,000 registered population. There were also significant reductions in gastrointestinal disease outcomes for vaccine-ineligible children aged 24–59 months. RVGE hospitalisations decreased in this age group by 66% (95% CI 44–81%) and all-cause AGE hospitalisations decreased by 26% (95% CI 11–39%). However, in the 2014/15 season, a peak of incidence was detected in May across all primary outcome measures, which was comparable in magnitude to the pre-vaccine rotavirus peak observed in March. Disease rates by surveillance year and pre- and post-vaccine introduction are provided in Additional file 1: Table S1.

#### Impact in children aged 5 to 14 years

In the pre-vaccine period, children aged 5–14 years had the lowest yearly rates of hospitalisation for all-cause AGE (18 per 10,000 population) (Table 2). Rotavirus seasonality in children aged 5–14 years was less pronounced and inconsistent across all outcome measures in the pre-vaccine period (Fig. 2). In this vaccine-ineligible age group, between July 2013 and June 2016 there were only two laboratory-confirmed detections of RVGE at Alder Hey Children’s Hospital. Furthermore, all-cause AGE hospitalisations and ED attendances for gastrointestinal conditions also fell (Table 2). GP consultations (-3%, 95% CI -21–12%) and WIC attendances (0%, 95% CI -52–34%) for infectious gastroenteritis remained similar to pre-vaccine levels. There were no differences between changes in incidence in the rotavirus season and out of the rotavirus season.

#### Impact in persons aged 15 to 64 years

**Fig. 3:**
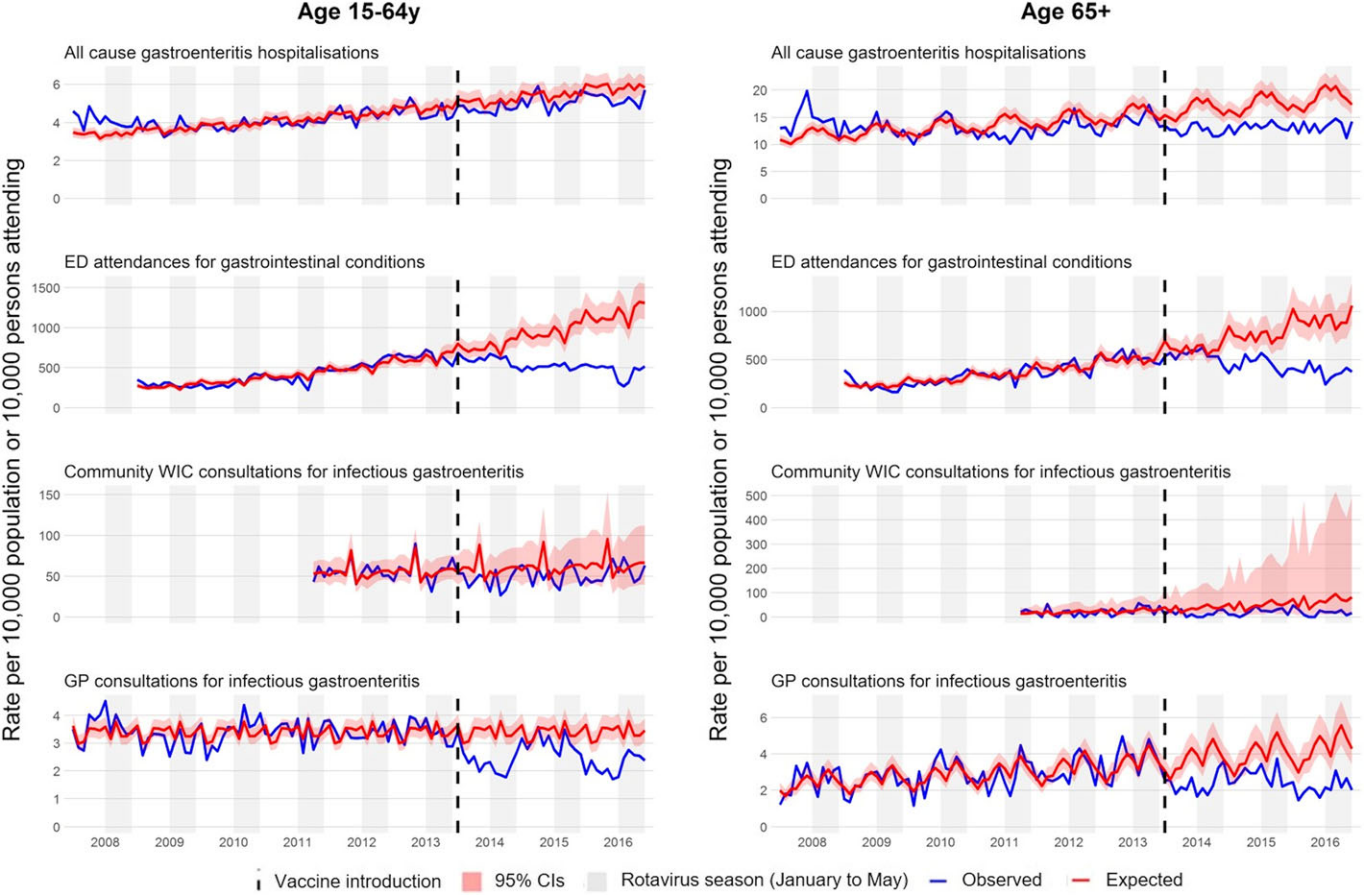
Trends in four study outcome measures for older children and adults aged 15+ years in Merseyside, UK, July 2008 to June 2016. Each analysis examines trends, including comparison of observed incidence (blue line) after rotavirus vaccination (July 2013 to June 2016) in the UK with expected incidence (red line) and associated 95% confidence intervals (red shaded area) in the absence of vaccination. Expected incidence and 95% confidence intervals are based on predictions from regression models fitted to available historic data for each outcome measure. The black hashed line represents the introduction of rotavirus vaccine in the UK in July 2013. CI confidence interval, ED emergency department, GP general practice, WIC walk-in centre

Data were available for four out of five of the primary outcomes. There was no clearly identified seasonality in the pre-vaccine period for the non-specific outcome measures in this age group (Fig. 3). Moderate reductions were seen in persons aged 15–64 years across all outcome measures (Table 2). In the post-vaccine period, hospitalisations for all-cause AGE fell by 8% (95% CI 2–14%), ED attendances for gastrointestinal conditions by 29% (95% CI 16–40), and WIC and GP consultations for infectious gastroenteritis by 24% (95% CI 7–38%) and 26% (95% CI 18–33%), respectively. There were no significant differences in the level of percentage change on comparing the in-season and out-of-season periods.

#### Impact in 65+ year olds

There were significant moderate reductions in all-cause AGE hospitalisations, ED attendances for gastrointestinal conditions and GP consultations for infectious gastroenteritis (Fig. 3 and Table 2). The reduction in attendance at WICs for infectious gastroenteritis was non-significant (47%; 95% CI -15–75%). The absolute rate of consultations averted was 19 per 10,000 registered population for GPs and 34 per 10,000 for WICs (Table 2). During the rotavirus season, proportional reductions were slightly higher than out-of-season, although the difference was not significant.

### Vaccine impact by socioeconomic deprivation status

#### Burden of gastrointestinal infection prior to vaccine introduction

**Fig. 4:**
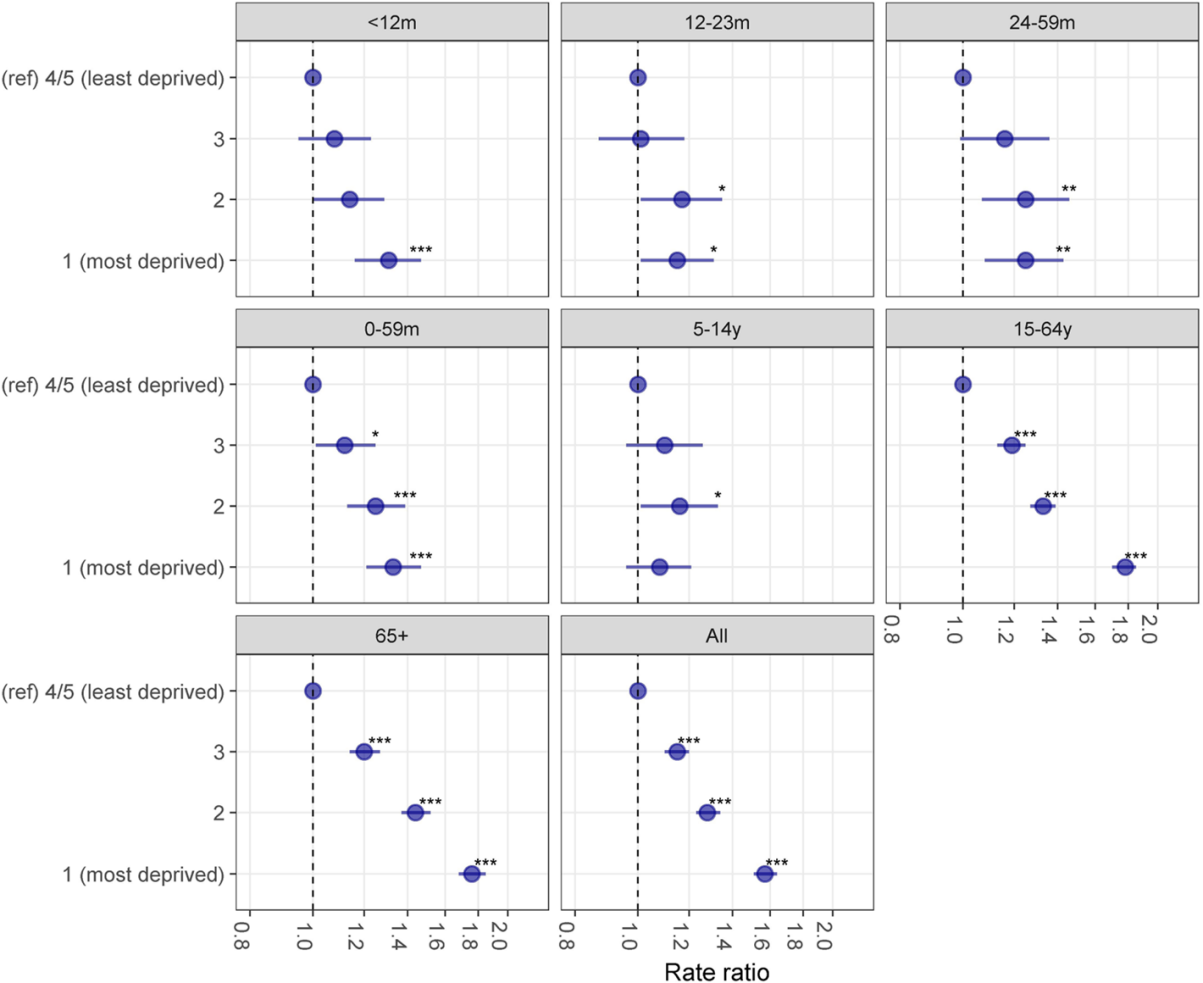
Relative risk of hospitalisation with acute all-cause-gastroenteritis prior to vaccine introduction, by age group and deprivation quintile, July 2004 to June 2013, Merseyside, UK. ref reference

Prior to vaccine introduction, the risk of being admitted to hospital for all-cause AGE was 57% higher (IRR = 1.57; 95% CI 1.51–1.64) in the most socioeconomically deprived populations of Merseyside compared to the least (Fig. 4). Age-group-stratified analyses showed that in all age groups apart from those 5–14 years of age (IRR = 1.08; 95% CI 0.96–1.21), the risk of hospitalisation with all-cause AGE was significantly greater in the most socioeconomically deprived populations of Merseyside compared to the least. Children < 12 months of age in the most socioeconomically deprived quintile had the highest rate of hospitalisation (47 per 1,000 person years), compared with 36 per 1,000 person years in the least deprived (IRR = 1.31; 95% CI 1.16–1.47). Among 12–23-month-olds, the age group with the second highest rates of hospitalisation, the difference between the most deprived (30 per 1,000 person years) and least deprived (26 per 1,000 person years) was less pronounced (IRR = 1.15; 95% CI 1.01–1.31).

#### Hospitalisations averted per child vaccinated

**Fig. 5:**
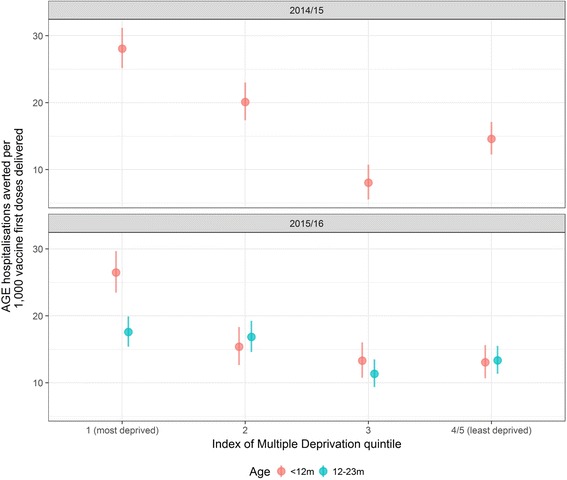
Estimated all-cause acute gastroenteritis hospitalisations averted per 1,000 vaccine first doses delivered in the 2014/15 and 2015/16 seasons for vaccine-eligible cohorts aged < 12 months and 12–23 months. AGE acute gastroenteritis

We estimated the number of all-cause AGE hospitalisations potentially averted in Merseyside due to rotavirus vaccination in two vaccine-eligible age cohorts, < 12 months and 12–23 months of age. In children aged < 12 months living in the most deprived populations, it was estimated that in 2014/15 and 2015/16, 28 (95% CI 25–31) and 26 (95% CI 23–30) all-cause AGE hospitalisations were averted per 1,000 first-dose rotavirus vaccines delivered, respectively. In the least deprived populations, 15 (95% CI 12–17) and 13 (95% CI 11–16) all-cause AGE hospitalisations were averted per 1,000 first-dose rotavirus vaccines delivered in 2014/15 and 2015/16, respectively (Fig. 5). For the cohort aged 12–23 months, it was estimated that there were 18 (95% CI 15–20) all-cause AGE hospitalisations averted per 1,000 persons vaccinated with at least one dose of rotavirus vaccine in 2015/16 in the most deprived populations, and 13 all-cause AGE hospitalisations averted (95% CI 11–16) in the least deprived populations.

If the WHO target for primary childhood immunisations of 95% uptake was attained in each deprivation stratum nationally (England), 10,811 all-cause AGE hospitalisations of infants would have been averted in 2015/16, with 41% (4,395; 95% CI 3,898–4,925) of those averted in the most deprived population (Table 3). Among 12–23-month-olds, 9,472 all-cause AGE hospitalisations would be expected to have been averted, with 31% (2,940; 95% CI 2,570–3,330) of those averted in the most deprived population.

**Table 3:** Predicted all-cause acute gastroenteritis hospitalisations averted nationally in children under 2 years of age in 2015/16 at 95% vaccine uptake

Age group	Index of Multiple Deprivation quintile	Estimated national population (2016) [16]	Hospitalisations averted at 95% vaccine uptake		
			Number	95% Lower CI	95% Upper CI
< 12 months	1 (most deprived)	174,784	4395	3898	4925
	2	149,462	2185	1795	2603
	3	126,372	1597	1292	1924
	4/5 (least deprived)	212,359	2634	2156	3147
	Total	662,977	10,811		
12–23 months	1 (most deprived)	176,129	2941	2579	3330
	2	149,862	2397	2080	2740
	3	126,517	1363	1124	1621
	4/5 (least deprived)	218,485	2771	2359	3218
	Total	670,993	9472		

*CI* confidence interval

## Discussion

In this study, which is one of the few studies to evaluate the impact of rotavirus vaccine introduction simultaneously across all levels of the health-care system in a defined geographic area, we have demonstrated reductions in gastrointestinal disease burden across all levels of health-care and across all ages. Reductions were greatest for the most specific and severe disease outcomes (rotavirus hospitalisations and AGE hospitalisations) during the rotavirus season and for the youngest children who were in vaccine-eligible age groups. Smaller reductions among older unvaccinated populations suggest herd protection. The impact of vaccination was also greater in the most socioeconomically deprived populations, despite lower vaccine coverage.

Most previous studies that evaluated rotavirus vaccine impact in high-income countries focussed on severe disease outcomes, with the magnitude of reductions similar to those described here for children in both vaccine-eligible and ineligible age groups [12, 29–42]. The reduction in all-cause AGE of 46% (36–55%) for infants and 50% (38–60%) for children 12–23 months of age was also similar to that reported in earlier UK studies, as was the indication of herd protective effects in older adults and children [12, 36].

For less severe disease outcomes (people with disease presenting to GPs and WICs), we demonstrated smaller relative reductions compared to more specific or severe disease outcomes. However, these reductions constitute a substantial contribution to the absolute number of health-care contacts averted through vaccination. The impact on non-specific outcome measures was consistently highest during the rotavirus season for children under 5 years, suggesting that the observed reduction in incidence of AGE is likely to be due to a real reduction in incidence of rotavirus disease. The smaller reductions seen in consultations in primary care (WICs and GPs) are likely explained by the non-specific gastroenteritis outcome measure and also because of the presumed lower effectiveness of rotavirus vaccine against milder disease [43, 44]. Furthermore, the reductions in GP consultations for infectious gastroenteritis observed for children in vaccine-eligible age groups (19% for infants and 13% for 12–23 months) are epidemiologically plausible, since a study from the pre-vaccine period estimated that rotavirus was detected by enzyme-linked immunosorbent assay (ELISA) in 14% and by ELISA and/or PCR in approximately 19% of infectious intestinal disease cases seen in GP consultations for UK children under 5 [45, 46], with this estimate likely to be higher in infants. Furthermore, the estimated reductions in WIC attendances and GP consultations are comparable to that reported from an analysis of UK syndromic surveillance of GP consultations for gastroenteritis, diarrhoea and vomiting (26% reduction for infants) [12]. They are also comparable with reductions in AGE outpatient attendances reported in Finland (13% reduction in infants) [33, 47] and all-cause AGE community clinic visits in Israel (19% reduction in infants and 16% for 12–23-month-olds) [48].

We have shown that the most deprived populations were at the greatest risk of all-cause AGE prior to vaccine introduction, with the highest rates of disease occurring in infants in the most deprived populations. This supports previous findings from a lower-resolution national study, which showed that the rate of hospitalisation with all-cause AGE increased with increasing deprivation [17]. The uptake of rotavirus vaccination in our study population was also associated with neighbourhood-level deprivation, with a significantly lower uptake of the first dose of the vaccine and lower completion of the full two-dose schedule in the most deprived populations. Similar findings have been shown in Merseyside for measles, mumps and rubella vaccination, and locally and nationally for childhood influenza vaccination [18, 19].

We were able to overlay a combination of small-area-level deprivation status, vaccine uptake and all-cause AGE hospitalisations to estimate the disease averted per first vaccine dose delivered for different deprivation strata. In infants, disease averted by vaccination was higher in the most deprived areas, suggesting that even with lower vaccine uptake, the most deprived populations benefit the most from the vaccination programme. The higher rates of disease averted in infants < 12 months of age living in the most deprived populations is likely to reflect the higher baseline burden of disease in this group and the relative inequity of hospitalisation rates prior to vaccine introduction. However, for 12–23-month-olds, there is a smaller difference in incidence of disease averted between the least deprived and the most deprived areas, reflecting the lower baseline inequity in disease burden between the deprivation strata.

Nationally, there are disproportionately more infants and young children living in the most deprived quintile (26%) compared to the least deprived (15%) [15, 16]. With individual-level vaccine effectiveness known to be lower in persons with a lower socioeconomic status from studies conducted in high-income settings [49, 50], improving vaccine uptake in the most deprived populations will have the biggest impact towards reducing rotavirus-associated disease. We estimate that over 41% of all-cause AGE hospitalisations averted in infants due to rotavirus vaccination would be averted in the most deprived populations if vaccine uptake was equitable across deprivation strata at the WHO vaccine uptake target of 95% [27, 28].

### Strengths and limitations

This ecological study using routine health service data is subject to a number of limitations. There is an inherent problem with clinical coding of rotavirus gastroenteritis in UK hospitals. A quality analysis at Alder Hey Children’s Hospital showed that only 39% of laboratory-confirmed rotavirus hospitalisations were coded as ICD-10 rotaviral enteritis (A08.0), and this figure is lower in other UK hospitals [51]. Therefore, in this study, for the RVGE hospitalisation outcome measure, we used hospitalisations that were laboratory-confirmed rotavirus from Alder Hey rather than ICD-10 codes.

In the context of this outcome measure, it is important to acknowledge the change in rotavirus diagnostic testing methods that occurred at Alder Hey during the study period (Table 1). An enzyme immunoassay was used for 10 of the 14 study years, whilst immuno-chromatography was utilised between 2005 and 2008. The immuno-chromatographic method used (VIKIA®, Rota-Adeno) has a slightly lower diagnostic accuracy compared to enzyme immunoassay methods [52, 53]. However, the pre-vaccine introduction time series spanned 11 years, and since the change in testing practices was not accompanied by a clear non-secular variation in RVGE hospitalisation rates, we would not expect this change to have impacted significantly on effect estimates.

Since rotavirus detection is not routinely undertaken in community settings, such as GP and WICs, syndromic and non-specific outcomes related to gastroenteritis were used, and we were, therefore, unable to account for the contribution of other pathogens causing AGE. However, the predictable seasonality of rotavirus infection allowed the analysis to focus on the rotavirus season, which should improve the robustness of reduction estimates in age-eligible children. In older children and adults, the estimates are more uncertain because there is limited laboratory testing and surveillance data on rotavirus seasonality and disease burden in these age groups in the pre-vaccine period. The lack of routine testing is evidenced by the recommendation in the *Standards for Microbiology Investigation S7: gastroenteritis and diarrhoea* that rotavirus testing is only standard for sporadic cases of gastroenteritis under the age of 5 years and immunocompromised cases [54].

Because of these limitations, the model fit was less good for older populations due to the less seasonal and more random incidence of gastroenteritis disease, and in these situations the analysis may have overestimated the impact of vaccination. Furthermore, we used a non-dynamic regression fit and so we did not account for changes in the force of the infection due to a reduction in the number of cases. We were, therefore, not able to adjust the predicted incidence to account for current levels of infection. A full transmission model would be required to describe fully the reduction in the transmission rate and associated case reduction due to vaccination. Despite these limitations, studies in the UK, Australia, Europe and the US also show an impact in older populations [12, 36, 37, 42, 55–57]. The number of hospitalisations averted nationally under a uniform 95% vaccine uptake was made using two main assumptions. Firstly, that the population of Merseyside is representative of the national population and secondly, that the relationship between vaccine uptake and the herd protective effect of vaccination is linear. Therefore, the estimates are likely to be conservative as a consequence of assuming a linear relationship, particularly if the level of rotavirus vaccine uptake required for population protection is reached before 95% uptake.

Finally, the novelty of measuring vaccine impact on multiple levels of a health system simultaneously in a defined population provides robustness that any detected changes are due to rotavirus vaccination rather than idiosyncrasies of one particular data set. For example, we detected delayed peak activity (April/May) in children aged 24–59 months across all outcome measures in season 2014/15, strengthening the evidence that the data sets used in this study were useful in detecting rotavirus activity in non-specific outcomes. This delayed peak is also observed in laboratory-confirmed rotavirus detections nationally.

## Conclusion

This analysis identified the effect of rotavirus vaccination on health-care utilisation for acute gastroenteritis in the four major levels of the UK health system for five outcomes of varying specificity. The study strongly indicates that rotavirus vaccination has reduced the incidence of acute gastroenteritis across the health-care system in both vaccine-eligible and ineligible populations. Rotavirus vaccination will, therefore, contribute to alleviating the increasing pressures on acute services across a health system. With an impact greater than that predicted through cost-effective modelling in the UK [58], these data strongly support the sustained use of the vaccine in the UK and continued expansion to other European countries.

We have also shown that prioritising vaccine uptake in the most socioeconomically deprived communities is likely to give the greatest health benefit in terms of population disease burden and can contribute to reducing health inequalities. Further studies are required to disentangle which factors related to socioeconomic deprivation have the greatest influence on vaccine acceptance, so that interventions to improve vaccine uptake can be targeted effectively.

## Additional file

Additional file 1: Table S1. Yearly rates of hospitalisation/attendance for different levels of the health system pre- and post-rotavirus vaccine introduction in Merseyside, UK. (DOCX 25 kb)

## Abbreviations

AGE: Acute gastroenteritis
CCG: Clinical commissioning group
CHIS: Child Health Information Service
CI: Confidence interval
ED: Emergency department
ELISA: enzyme-linked immunosorbent assay
GP: General practice
ICD: International Classification of Diseases
IMD: Index of Multiple Deprivation
IRR: Incidence rate ratio
LSOA: Lower super output area
NHS: National Health Service
RR: risk ratio
RVGE: Rotavirus gastroenteritis
WHO: World Health Organisation
WIC: walk-in centre
