# Supplementary material for: Rotavirus vaccine impact and socioeconomic deprivation: an interrupted time-series analysis of gastrointestinal disease outcomes across primary and secondary care in the UK
Source: BMC Med. 2018 Jan 29;16:10. doi: 10.1186/s12916-017-0989-z (PMC5787923; doi:10.1186/s12916-017-0989-z)
Supplement: Additional file 1: Table S1. — Yearly rates of hospitalisation/attendance for different levels of the health system pre- and post-rotavirus vaccine introduction in Merseyside, UK. (DOCX 25 kb) [file 12916_2017_989_MOESM1_ESM.docx]

**Table S1: Yearly rates of hospitalisation / attendance for different levels of the health system pre- and post-rotavirus vaccine introduction in Merseyside, UK**

| **Age group** | **Yearly rate of hospitalisation / attendance (per 10,000)^¥^** | | | | | | | | | | | | | | | |
| --- | --- | --- | --- | --- | --- | --- | --- | --- | --- | --- | --- | --- | --- | --- | --- | --- |
|  | **Pre-vaccine Introduction** | | | | | | | | | | | | | **Post-vaccine introduction** | | |
|  | **2000/01** | **2001/02** | **2002/03** | **2003/04** | **2004/05** | **2005/06** | **2006/07** | **2007/08** | **2008/09** | **2009/10** | **2010/11** | **2011/12** | **2012/13** | **2013/14** | **2014/15** | **2015/16** |
| **Hospitalisations for laboratory confirmed rotavirus to Alder Hey** | | | | | | | | | | | | | | | | |
| **<12m** | - | - | 158 | 168 | 148 | 182 | 85 | 123 | 85 | 113 | 125 | 130 | 168 | 16 | 23 | 4 |
| **12-23m** | - | - | 159 | 118 | 145 | 157 | 88 | 140 | 97 | 130 | 136 | 112 | 102 | 30 | 19 | 0 |
| **24-59m** | - | - | 44 | 36 | 30 | 48 | 28 | 42 | 25 | 33 | 23 | 26 | 40 | 5 | 17 | 7 |
| **5-14y** | - | - | 7 | 6 | 5 | 8 | 6 | 9 | 8 | 9 | 11 | 6 | 6 | 1 | 1 | 0 |
| **Total 0-59m** | - | - | 106 | 98 | 93 | 121 | 62 | 94 | 63 | 84 | 84 | 82 | 96 | 14 | 19 | 5 |
| **Hospitalisations for all cause acute gastroenteritis** | | | | | | | | | | | | | | | | |
| **<12m** | 351 | 322 | 312 | 353 | 285 | 387 | 563 | 577 | 492 | 580 | 363 | 298 | 328 | 249 | 222 | 220 |
| **12-23m** | 251 | 227 | 233 | 231 | 209 | 259 | 335 | 343 | 295 | 404 | 244 | 227 | 254 | 138 | 124 | 123 |
| **24-59m** | 68 | 63 | 68 | 55 | 58 | 63 | 98 | 94 | 91 | 98 | 63 | 51 | 63 | 42 | 67 | 53 |
| **5-14y** | 14 | 13 | 12 | 13 | 13 | 17 | 25 | 25 | 25 | 28 | 22 | 16 | 20 | 18 | 19 | 22 |
| **15-64y** | 30 | 27 | 27 | 25 | 29 | 32 | 44 | 49 | 45 | 46 | 48 | 53 | 55 | 56 | 61 | 63 |
| **65+** | 82 | 87 | 111 | 101 | 117 | 120 | 169 | 173 | 158 | 155 | 140 | 160 | 172 | 151 | 159 | 159 |
| **Total 0-59m** | 156 | 143 | 147 | 148 | 135 | 169 | 243 | 247 | 217 | 260 | 161 | 137 | 158 | 103 | 109 | 100 |
| **ED attendances for gastrointestinal conditions (no admission)** | | | | | | | | | | | | | | | | |
| **<12m** | - | - | - | - | - | - | - | - | 1654 | 1889 | 2101 | 2280 | 2240 | 1959 | 2115 | 1610 |
| **12-23m** | - | - | - | - | - | - | - | - | 978 | 1003 | 1102 | 1314 | 1340 | 942 | 1050 | 758 |
| **24-59m** | - | - | - | - | - | - | - | - | 650 | 691 | 750 | 828 | 887 | 800 | 945 | 604 |
| **5-14y** | - | - | - | - | - | - | - | - | 406 | 456 | 584 | 656 | 711 | 698 | 712 | 601 |
| **15-64y** | - | - | - | - | - | - | - | - | 286 | 304 | 375 | 525 | 630 | 606 | 507 | 432 |
| **65+** | - | - | - | - | - | - | - | - | 237 | 278 | 350 | 404 | 521 | 545 | 443 | 366 |
| **Total 0-59m** | - | - | - | - | - | - | - | - | 1022 | 1105 | 1239 | 1395 | 1425 | 1180 | 1332 | 948 |
| **Walk-in centre attendances for infectious gastroenteritis*** | | | | | | | | | | | | | | | | |
| **<12m** | - | - | - | - | - | - | - | - | - | - | - | 604 | 580 | 369 | 378 | 371 |
| **12-23m** | - | - | - | - | - | - | - | - | - | - | - | 468 | 488 | 249 | 325 | 206 |
| **24-59m** | - | - | - | - | - | - | - | - | - | - | - | 209 | 184 | 144 | 176 | 143 |
| **5-14y** | - | - | - | - | - | - | - | - | - | - | - | 81 | 78 | 66 | 89 | 60 |
| **15-64y** | - | - | - | - | - | - | - | - | - | - | - | 56 | 56 | 45 | 52 | 54 |
| **65+** | - | - | - | - | - | - | - | - | - | - | - | 15 | 29 | 16 | 20 | 19 |
| **Total 0-59m** | - | - | - | - | - | - | - | - | - | - | - | 380 | 361 | 223 | 262 | 211 |
| **GP consultations for infectious gastroenteritis** | | | | | | | | | | | | | | | | |
| **<12m** | - | - | - | - | - | - | - | 712 | 678 | 677 | 697 | 625 | 658 | 452 | 524 | 500 |
| **12-23m** | - | - | - | - | - | - | - | 692 | 567 | 652 | 500 | 558 | 582 | 335 | 488 | 429 |
| **24-59m** | - | - | - | - | - | - | - | 183 | 187 | 209 | 178 | 164 | 182 | 125 | 209 | 164 |
| **5-14y** | - | - | - | - | - | - | - | 56 | 49 | 55 | 56 | 50 | 52 | 46 | 62 | 60 |
| **15-64y** | - | - | - | - | - | - | - | 43 | 37 | 41 | 41 | 40 | 41 | 28 | 36 | 26 |
| **65+** | - | - | - | - | - | - | - | 30 | 30 | 35 | 34 | 36 | 44 | 28 | 33 | 26 |
| **Total 0-59m** | - | - | - | - | - | - | - | 391 | 361 | 391 | 346 | 335 | 357 | 232 | 328 | 284 |

^¥^Table 1 provides specific denominators for each outcome measure

*2010/11 not included because data were only available for a part of the year.
